# Mutation in *flrA* and *mshA* Genes of *Vibrio cholerae* Inversely Involved in *vps*-Independent Biofilm Driving Bacterium Toward Nutrients in Lake Water

**DOI:** 10.3389/fmicb.2017.01770

**Published:** 2017-09-13

**Authors:** Shrestha Sinha-Ray, Afsar Ali

**Affiliations:** ^1^Emerging Pathogens Institute, University of Florida, Gainesville FL, United States; ^2^Department of Microbiology and Cell Science, College of Agricultural and Life Sciences, University of Florida, Gainesville FL, United States; ^3^Department of Environmental and Global Health, College of Public Health and Health Professions, University of Florida, Gainesville FL, United States

**Keywords:** *Vibrio cholerae*, biofilm, motility, nutrient-poor, microcosm, chemotaxis

## Abstract

Many bacterial pathogens promote biofilms that confer resistance against stressful survival conditions. Likewise *Vibrio cholerae* O1, the causative agent of cholera, and ubiquitous in aquatic environments, produces *vps*-dependent biofilm conferring resistance to environmental stressors and predators. Here we show that a 49-bp deletion mutation in the *flrA* gene of *V. cholerae* N16961S strain resulted in promotion of *vps-*independent biofilm in filter sterilized lake water (FSLW), but not in nutrient-rich L-broth. Complementation of *flrA* mutant with the wild-type *flrA* gene inhibited *vps*-independent biofilm formation. Our data demonstrate that mutation in the *flrA* gene positively contributed to *vps*-independent biofilm production in FSLW. Furthermore, inactivation of *mshA* gene, encoding the main pilin of mannose sensitive hemagglutinin (MSHA pilus) in the background of a Δ*flrA* mutant, inhibited *vps-*independent biofilm formation. Complementation of Δ*flrA*Δ*mshA* double mutant with wild-type *mshA* gene restored biofilm formation, suggesting that *mshA* mutation inhibited Δ*flrA*-driven biofilm. Taken together, our data suggest that *V. cholerae flrA* and *mshA* act inversely in promoting *vps*-independent biofilm formation in FSLW. Using a standard chemotactic assay, we demonstrated that *vps*-independent biofilm of *V. cholerae*, in contrast to *vps*-dependent biofilm, promoted bacterial movement toward chitin and phosphate in FSLW. A Δ*flrA*Δ*mshA* double mutant inhibited the bacterium from moving toward nutrients; this phenomenon was reversed with reverted mutants (complemented with wild-type *mshA* gene). Movement to nutrients was blocked by mutation in a key chemotaxis gene, *cheY*-3, although, *cheY*-3 had no effect on *vps*-independent biofilm. We propose that in fresh water reservoirs, *V. cholerae*, on repression of flagella, enhances *vps*-independent biofilm that aids the bacterium in acquiring nutrients, including chitin and phosphate; by doing so, the microorganism enhances its ability to persist under nutrient-limited conditions.

## Introduction

*Vibrio cholerae*, toxigenic strains of which cause epidemic cholera, is ubiquitous to aquatic environment. Ingestion of water and/or food contaminated with the microorganism leads to cholera (Colwell and Huq, 1994; Kaper et al., 1995; Faruque et al., 1998; Morris, 2011). In aquatic reservoirs, *V. cholerae* can persist either in a planktonic (free-living) stage or in biofilms (Tamplin et al., 1990; Shukla et al., 1995). In the biofilm mode of persistence, the microorganism can attach to biotic and abiotic surfaces, including phytoplankton, crustaceans, zooplanktons, aquatic plants, sediments, and detritus (Huq et al., 1983; Huq et al., 1990; Islam et al., 1990; Nelson et al., 2009). Despite decades of investigations, the physiological and genetic basis of the persistence of *V. cholerae* in aquatic reservoirs, particularly during inter-epidemic periods, remains to be fully elucidated. Possible survival mechanisms in nutrient-poor and stressful aquatic reservoirs include: (a) adopting a viable but non-culturable (VBNC) state (Xu et al., 1982; Colwell and Huq, 1994), (b) switching from planktonic to biofilm lifestyle (Wai et al., 1998; Yildiz and Schoolnik, 1999; Ali et al., 2002), and/or (c) assuming “persister” and “growth advantage stationary phase” (“GASP”) phenotypes as described recently (Jubair et al., 2012, 2014).

*Vibrio cholerae* produces a single polar flagellum that contributes to motility during the planktonic stage of growth, but is inhibited in mature biofilm growth stage. Biogenesis of flagella is encoded by four gene clusters. Gene cluster I harbors a master transcriptional activator, FlrA encoded by the *flrA* gene. Coupled with δ^54^, FlrA promotes transcription of other flagellar genes present in clusters I–IV; furthermore, FlrA also contributes to the transcription of a number of genes required for chemotaxis (Klose and Mekalanos, 1998) (see below). During environmental persistence, *V. cholerae* employs its flagella in two distinct ways: first, flagella promote planktonic bacteria to propel to a growth-permissible environment, while they drive back the bacteria in response to non-permissible and hostile growth conditions. Secondly, flagella play a pivotal role in sensing and attaching to biotic or abiotic surfaces triggering the initial steps of biofilm formation that ultimately lead to mature biofilm (Watnick and Kolter, 1999; Butler and Camilli, 2005). Interestingly, in a mature biofilm, *V. cholerae* represses flagella synthesis while it promotes exopolysaccharide production, a key ingredient for mature biofilm formation (Watnick and Kolter, 1999; Prouty et al., 2001). However, it is worth noting here that many bacterial species, including *Acinetobacter baumannii* (Tomaras et al., 2008; Gaddy and Actis, 2009) do not possess flagella, yet they are able to produce biofilm suggesting that flagella may be dispensable in biofilm formation in non-motile bacteria.

In addition to flagella, *V. cholerae* produces two types of pili, including a mannose-sensitive hemagglutinin (MSHA) pilus (also known as type IVa pilus), and a toxin co-regulated pilus (TCP) (also known as type IVb pilus). The main pilin of MSHA and TCP pili is encoded by *mshA* and *tcpA* gene, respectively. While MSHA pilus promotes strong surface attachment facilitating biofilm formation and environmental persistence (Watnick et al., 1999; Hsiao et al., 2009), TCP pilus contributes to virulence by allowing the bacterium to attach to and colonize human intestinal epithelial cells (Herrington et al., 1988; Rhine and Taylor, 1994; Hsiao et al., 2008). Interestingly, biogenesis of the two pili is promoted by opposite conditions. For example, TCP pilus is highly expressed in the human intestine with repression of MSHA pilus. In contrast, MSHA pilus is profusely expressed under *in vitro* conditions, resulting in biofilm formation with repression of TCP pilus (Hsiao et al., 2008, 2009).

In many bacterial species, chemotaxis promotes the ability of motile bacteria to swim toward or away from specific environmental stimuli in order to provide the microorganism with a survival advantage in a given condition (Wadhams and Armitage, 2004; Butler and Camilli, 2005). Chemotaxis has been extensively investigated in *Escherichia coli* and *Salmonella typhimurium* (Bourret and Stock, 2002; Wadhams and Armitage, 2004). In contrast to *E. coli* or *S. typhimurium*, multiple paralogs of various chemotaxis genes are present in *V. cholerae* chromosomes (Heidelberg et al., 2000). With the exception of the *mcp* gene encoding methyl-accepting chemotaxis protein, which is scattered throughout the genome, most *V. cholerae* chemotaxis genes are organized into three operons (Heidelberg et al., 2000; Butler and Camilli, 2005). Only one of these three operons, operon 2, is important for chemotaxis in *V. cholerae* as genes present in this operon promoted chemotaxis (Butler and Camilli, 2005). Previous studies demonstrated that *cheA*-2 (Gosink et al., 2002) and *cheY*-3 (Lee et al., 2001) genes of operon 2 were required for chemotaxis, whereas *cheA*-1 and *cheY*-1, and *cheA*-3 and *cheY*-3 from operons 1 and 3, respectively, were dispensable for chemotaxis (Heidelberg et al., 2000).

Long-term persistence of bacteria in stressful and stationary growth phase can promote a “GASP” phenotype (Finkel and Kolter, 1999; Zinser and Kolter, 2004; Finkel, 2006). We recently reported a *V. cholerae* GASP phenotype (GASP-700D) after its long-term persistence (700 days) in nutrient-poor lake water microcosms (Jubair et al., 2014). Compared to its wild-type N16961S phenotype, the GASP-700D phenotype was defective in motility and produced significantly increased *vps*-independent biofilm when grown specifically in filter sterilized lake water (FSLW). Given the non-motile behavior and ∼1000-fold down regulation of *flrA* gene of GASP-700D, we hypothesized that GASP-700D sustained a mutation in the *flrA* gene contributing to increased expression of *vps*-independent biofilm. Here we provide evidence that: (a) *flrA* gene in GASP-700D sustained a 49-bp internal deletion mutation resulting in an increased production of *vps*-independent biofilm in FSLW, but not in nutrient-rich L-broth, (b) mutation in the *mshA* gene inhibited the *vps*-independent biofilm formation, (c) *vps*-independent, in contrast to *vps*-dependent biofilm, promoted biofilm bacterium to move toward nutrients, including chitin and phosphate in FSLW, and (d) mutation in the chemotaxis gene, *cheY-3*, inhibited the mutant from moving toward nutrients in FSLW.

## Materials and Methods

### Bacterial Strains and Growth Conditions

Bacterial strains and plasmids used in this study are listed in **Table 1**. In this study, as needed, we used two variants of *V. cholerae* N16961 strain, including N16961S (stands for smooth variant) and N16961R (stands for “rugose” variant). The method used to convert smooth variant to rugose variant has been described previously (Ali et al., 2002). As needed, bacterial strains were subcultured from glycerol stock stored at -80°C onto L-agar and the culture was incubated overnight at 37°C. For growth in L-broth, a single colony was transferred to 3 ml L-broth and the culture was grown overnight at 37°C with a shaking speed of 250 × *g* in an environmental shaker (New Brunswick Scientific, Edison, NJ, United States). Unless otherwise indicated, for culture in FSLW, 1 ml of overnight L-broth grown culture was centrifuged, washed 2× in 1 ml FSLW and finally the pellet was resuspended in 1 ml fresh FSLW. As needed, antibiotics were used at the following concentrations: ampicillin, 100 μg ml^-1^ and polymyxin B, 50 U ml^-1^.

**Table 1:** Bacterial strains and plasmids.

Strain or plasmid	Description^a^	Reference
***V. cholerae* strains**		
N16961S	A wild-type, smooth, O1 El Tor strain isolated in Bangladesh in 1971	Ali et al., 2002
N16961R	A rugose variant of N16961S strain	Ali et al., 2002
GASP-700D (referred here as GSΔ*flrA*)	700 days old N16961S culture persisting in nutrient-poor FSLW was grown on L-agar and subsequently stored in FSLW supplemented with 30% glycerol at -80°C	Jubair et al., 2014
AAS80 (GSΔ*flrA*/pAA146)	The above strain was transformed with pAA146, carrying wild-type *flrA* gene cloned into pWSK29 (complementing vector)	This study
AAS84 (N16961SΔ*flrA*)	A 49-bp *flrA* internal deletion mutation created in the background of N16961S strain	This study
AAS81 (N16961SΔ*flrA*/pAA146)	The strain AAS84 was transformed with pAA146, carrying wild-type *flrA* gene cloned into pWSK29 (complementing vector)	This study
AA296 (GSΔ*flrA*Δ*mshA*)	A Δ*mshA* (309-bp in-frame deletion) created in the background of GSΔ*flrA* strain	This study
AA297 (GSΔ*flrA*Δ*mshA* /pAA159)	The strain AA296 was transformed with pAA159, carrying wild-type *mshA* gene cloned into pMMB67EH (complementing vector)	This study
AAS78 (N16961SΔ*mshA)*	A Δ*mshA* (309-bp in-frame deletion) created in the background of N16961S strain	This study
AAS79 (N16961SΔ*mshA*/pAA159)	The strain AAS78 was transformed with pAA159, carrying wild-type *mshA* gene cloned into pMMB67EH (complementing vector)	This study
AA218 (N16961SΔ*vpsA*)	A Δ*vpsA* in-frame null mutation created in the background of N16961S strain	Jubair et al., 2014
AA219 (N16961RΔ*vpsA*)	A Δ*vpsA* in-frame null mutation created in the background of N16961R strain	Jubair et al., 2014
AA220 (GSΔ*flrA*Δ*vpsA*)	A Δ*vpsA* in-frame null mutation created in the background of GSΔ*flrA* strain	Jubair et al., 2014
AA282 (GSΔ*flrA*Δ*cheY-3*)	A Δ*cheY-3* (406-bp null deletion) created in the background of GSΔ*flrA* strain	This study
AA284 (GSΔ*flrA*Δ*cheY-3*/pAA153)	The strain AA282 was transformed with pAA153, carrying wild-type *cheY-3* gene cloned into pWSK29 (complementing vector)	This study
AA294 (N16961SΔ*flrA*Δ*cheY-3*)	A Δ*cheY-3* (406-bp null deletion) created in the background of N16961SΔ*flrA* strain	This study
AA295 (N16961SΔ*flrA*Δ*cheY-3*/pAA153)	The strain AA294 was transformed with pAA153, carrying wild-type *cheY-3* gene cloned into pWSK29 (complementing vector)	This study
AAS99 N16961SΔ*cheY-3*	A Δ*cheY-3* (406-bp null deletion) created in the background of N16961S strain	This study
***E. coli* strains**		
DH5α	*recA* Δ*lac*U169 φ80d *lacZ*ΔM15	Gibco, BRL
SM 10 λ *pir*	*thi thr leu tonA* Km^r^	Miller and Mekalanos, 1988
**Plasmids**		
pWSK29	Low-copy-number vector, Amp^r^, *ori* pSC101	Wang and Kushner, 1999
pCVD442	Suicide vector, *ori* R6K, Amp^r^, *sacB*	Donnenberg and Kaper, 1991
pAAS82	A 1421-bp PCR fragment (*SacI*–*SalI*) of *flrA* gene amplified from GSΔ*flrA* (with a 49-bp internal deletion of *flrA*) was cloned into similarly digested pCVD442, Amp^r^	This study
pAA146	A 1470-bp PCR fragment (*SacI*–*SalI*) of wild-type *flrA* gene amplified from N16961S was cloned into similarly digested pWSK29, Amp^r^ (complementing vector)	This study
pAAS77	A 837-bp PCR fragment (*SalI*–*SacI*) carrying a 309-bp null deletion of *mshA* was cloned into similarly digested pCVD442, Amp^r^	This study
pAA159	A 551-bp PCR fragment (*KpnI*–*SphI*) of wild-type *mshA* gene was cloned into similarly digested pMMB67EH, Amp^r^ (complementing vector)	Andrew Camilli Lab
pAA143	A 984-bp PCR fragment (*SacI*–*SalI*) carrying a 406-bp null deletion of *cheY-3* was cloned into similarly digested pCVD442, Amp^r^	This study
pAA153	A 1293-bp PCR fragment (*SacII*–*EcoR1*) of wild-type *cheY-3* gene amplified from N16961S was cloned into similarly digested pWSK29, Amp^r^ (complementing vector)	This study

*^a^Relevant antibiotic resistances are indicated by ^*r*^. Km, kanamycin; Amp, ampicillin.*

### Water Source

For this study we used fresh water collected in a sterile 1,000 ml Nalgene bottle (Nalgene, Rochester, NY, United States) from a 30.2-acre natural lake (Wauburg Lake) in Gainesville, FL, United states as described previously (Jubair et al., 2012). For experiment, aliquot (300 ml) of water was filter sterilized using Nalgene 0.22 μm membrane filter unit. Chemical analysis of filter sterilized lake water was performed by Advanced Environmental Laboratories, Inc. (Gainesville, FL, United States). The analysis of lake water revealed following major components: total organic carbon (12.0 mg/l), total nitrogen (1.4 mg/l), ammonia (0.06 mg/l), nitrate plus nitrite (0.014 mg/l), sodium (7.3 mg/l), chloride (14.0 mg/l), calcium (7.0 mg/l), iron (0.03 mg/l), potassium (0.93 mg/l), total phosphate (0.26 mg/l), orthophosphate (0.002 mg/l), and magnesium (1.6 mg/l). Interestingly, low nutrient composition found in Wauburg Lake water was similar to what has been reported by us using the same lake water (Jubair et al., 2012), and in pond water obtained from Bangladesh and other fresh waters obtained from United States (Kierek and Watnick, 2003a; Nelson et al., 2008).

### Genetic Manipulations

To determine if GASP-700D (Jubair et al., 2014) sustained a mutation in the *flrA* gene, we PCR amplified the gene from the GASP-700D genome with two convergent PCR primers [aa778 and aa780 (Supplementary Table 1)] using standard PCR conditions. The amplicon was purified using Qiagen PCR Purification Kit (Qiagen, Valencia, CA, United States) and sequenced with help from the Interdisciplinary Center for Biotechnology Research (ICBR) at University of Florida; sequence analysis revealed that GASP-700D sustained a 49-bp deletion mutation in *flrA* gene (we hereafter referred this strain as GSΔ*flrA*).

For creating an identical 49-bp deletion mutation in *flrA* gene in the background of wild-type *V. cholerae* N16961S strain (**Table 1**), a one-step PCR cloning strategy was employed using primers, including aa778S and aa780S (Supplementary Table 1) carrying *SalI* and *SacI* site at the 5′-end of each primer, respectively. Genomic DNA of GSΔ*flrA* was used as PCR template while primer pairs (aa778S and aa780S) flanking the 49-bp deletion in GSΔ*flrA* were used to amplify the PCR product using standard PCR conditions. The purified PCR product was digested with *SalI* and *SacI*, the digested product was purified, and ligated into a similarly digested suicide vector, pCVD442 (Donnenberg and Kaper, 1991); the ligated product was transformed into *E. coli* SM10 λ *pir* using standard transformation procedures resulting in plasmid pAAS82. *E. coli* SM10 λ *pir* carrying pAAS82 was conjugated to *V. cholerae* N16961S; selection of transconjugants and counter selection for the *flrA* mutation resulting from homologous recombination were performed as described previously (Jubair et al., 2014). PCR and DNA sequencing methodologies were used to confirm the 49-bp mutation in *flrA* gene [AAS84 (N16961SΔ*flrA*), **Table 1**]. An in-frame deletion in the Δ*mshA* gene was created in the backgrounds of N16961S and GSΔ*flrA* by using primers and methodologies described previously (Thelin and Taylor, 1996). A two-step PCR cloning strategy, with a set of targeted primers, was used to create a null mutation in *cheY-3* gene in the backgrounds of N16961S, N16961SΔ*flrA*, and GSΔ*flrA* strains. Mutation in *vpsA* gene (VC0917) encoding UDP-*N*-acetylglucosamine 2-epimerase (Ali et al., 2000b), a key component of *vps*-dependent biofilm in the background of *V. cholerae* N16961S, N16961R, and GSΔ*flrA* strains was created as described previously (Jubair et al., 2014). To complement mutants, each wild-type gene of interest was PCR amplified using two convergent PCR primers targeting that gene; chromosomal DNA of N16961S was used as the template for PCR reactions. Following purification of the PCR product, the gene was cloned into pWSK29 vector (Wang and Kushner, 1999). For complementation of Δ*mshA*, we used plasmid vector pMMB67EH carrying wild-type *mshA* gene cloned into the vector (complementation plasmid is a kind gift from Andrew Camilli of Tufts University) (**Table 1**). The complementing plasmid was introduced into the *V. cholerae* mutant strain of interest using DNA electroporation (Ali et al., 2000a). All PCR primers used either in gene disruptions or in cloning wild-type genes are listed in Supplementary Table 1.

### Motility Assay

Swarming behavior of *V. cholerae* strains on motility agar (0.3% agar) was examined as described previously (Gardel and Mekalanos, 1996). Briefly, each strain of interest was grown overnight in L-broth at 37°C with a shaking speed of 250 × *g*. A sterile inoculating wire was dipped into the culture and the wire containing the culture was then inoculated onto motility agar. Zone of migration of each bacterial strain around the inoculating site was examined and the image was captured after incubation of the microorganism in motility agar at 37°C for 8 h.

### Quantitative Biofilm Assay

Quantification of biofilm production by *V. cholerae* strains grown in L-broth was measured as described previously (Watnick and Kolter, 1999). For measurement of biofilm production in FSLW microcosm, a single colony of *V. cholerae* grown on L-agar was transferred into 3 ml L-broth and the culture was incubated overnight at 37°C with a shaking speed of 250 × *g*. One milliliter of culture was spun down and washed 2× with 1 ml of FSLW. A 24-well polystyrene plastic plate (Corning Incorporated, Corning, NY, United States) was used as the surface for bacterial attachment; 50 μl of culture was added to 450 μl of fresh FSLW (10-fold dilution) to obtain ca. 10^8^ cfu/ml in each well (six replicates). The culture was incubated overnight statically at ambient temperature. Following incubation, the culture was discarded from the wells and washed 2× with water to discard any residual bacterial cells. Crystal violet (CV) dye was added to each well and the plate was incubated at room temperature for 30 min; the CV dye was discarded and the well was washed (2×) with water. Finally, cell-associated CV dye was extracted with 600 μl of dimethyl sulfoxide (DSMO) (Sigma, St. Louis, MO, United States) and the quantitative biofilm production was determined as described previously (Jubair et al., 2014).

### Confocal Microscopy

In order to determine the three-dimensional architectural structure and thickness of the biofilm, confocal microscopic analysis was performed as described previously (Jubair et al., 2014). *V. cholerae* strains, including N16961S, N16961SΔ*flrA*, GSΔ*flrA*, GSΔ*mshA*, and GSΔ*mshA/*pAA159 (**Table 1**) were included in the assay. Briefly, a single colony of each strain was inoculated into 3 ml L-broth and the culture was incubated and processed as described above. An inoculum (ca. 10^8^ cfu/ml) was transferred into a well containing 600 μl of fresh sterile FSLW in a 12-well polystyrene culture plate (Thermo Scientific Nunc, Pittsburgh, PA, United States). To provide a bacterial attachment platform, a 12 mm round glass cover slip (Fischer Scientific, Pittsburgh, PA, United States) was dipped into each culture well, and the culture was incubated statically overnight at room temperature. The following day, attached cells on cover slip were washed twice with Dulbecco’s PBS (DPBS) (HyClone Laboratories, Logan, UT, United States), and fixed in 10% neutral buffered formalin solution (Sigma-Aldrich, St. Louis, MO, United States) for 30 min. Cells were washed again with DPBS and stained with 500 μl/well of 1:1000 SYTO 9 dye (LIVE/DEAD BacLight Bacterial Viability Kit; Invitrogen, Grand Island, NY, United States) as described previously (Jubair et al., 2014). Following two washes with HyPure cell culture grade water (HyClone Laboratories, Logan, UT, United States), the glass cover slip was mounted on a 75 × 25 mm microscopic slide (Corning Inc., Corning, NY, United States). A Zeiss LSM 800 with Axio Observer Z1 inverted microscope (Carl Zeiss Iberia, S.L., Spain) was used to visualize the cover slip with an excitation and emission wavelengths of 484 and 500 nm, respectively. The attached microorganism was examined using Plan-Apochromat 63× objective lens. At least three biological replicates were examined during image analysis. All images and *z*-stacks were processed using Zen 2 (Blue edition) software and enhanced by adjusting the histogram of the image.

### Chemotaxis Assays

A chemotaxis assay was performed as described previously (Butler et al., 2006) with slight modifications. Briefly, a single colony of *V. cholerae* strain was grown in 3 ml L-broth overnight at 37°C with a shaking speed of 250 × *g*. One milliliter of culture was centrifuged; after decanting supernatant, the pellet was washed 2× with fresh FSLW and resuspended in 1 ml fresh FSLW. The culture was diluted (10-fold) in FSLW to obtain ca. 10^8^ cfu/ml and incubated overnight statically at room temperature. An aliquot of 150 μl of culture (three replicates) was transferred into 96-well plate. One microliter capillary tube (55 mm) (Drummond Scientific Company, Broomall, PA, United States) filled with either FSLW or FSLW supplemented with chitin (0.05%) or K_2_HPO_4_ (1.0 mM at final concentration) was inserted into each culture well. After 6 min of incubation at room temperature, the content of the capillary tube was expelled out into 99 μl of L-broth. The culture was spread onto L-agar plates and the plates were incubated overnight at 37°C; total bacterial number (colony forming units per milliliter) grown on L-agar was determined by standard plate count. The results were expressed as a comparative index (CI) for each strain. CI was calculated by the number of bacterial counts (colony forming units per milliliter) present in FSLW in the capillary tube supplemented with nutrient divided by the number of bacterial counts (colony forming units per milliliter) present in FSLW in the capillary tube not supplemented with nutrient.

### Statistical Analysis

One-way ANOVA along with Holm-Sidak’s multiple comparison test was performed using GraphPad Prism version 6.00 for Windows, GraphPad Software, La Jolla, CA, United States, http://www.graphpad.com. A *p*-value of <0.05 was considered as statistically significant.

## Results

### GASP-700D Sustained an Internal Deletion Mutation

**FIGURE 1:**
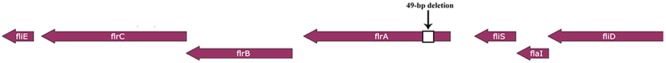
Schematic representation of open reading frame (ORF) of *flrA* gene. ORFs flanking *flrA* gene are drawn in the diagram with a black arrow indicating 49-bp deletion site in the *flrA* gene. ORFs are drawn to a scale using SnapGene software.

**FIGURE 2:**
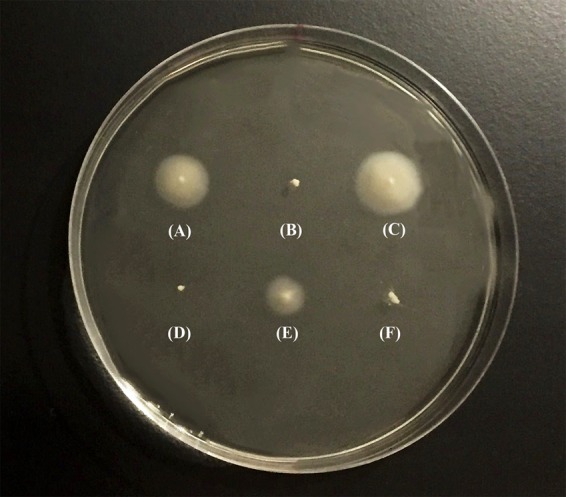
Swarming behavior of *V. cholerae* strains. Swarming behavior of *V. cholerae* strains was examined on motility agar (0.3% soft agar). Bacterial strains were individually grown overnight in L-broth at 37°C before inoculating them into motility agar. After inoculation, the plates were incubated at 37°C for 8 h and photograph of the culture plate was taken following incubation. **(A)** *V. cholerae* N16961S, **(B)** N16961SΔ*flrA*, **(C)** N16961SΔ*flrA*/pAA146, **(D)** GSΔ*flrA*, **(E)** GSΔ*flrA* /pAA146, and **(F)** N16961SΔ*flaA*.

After PCR amplification and sequencing of the *flrA* gene of GASP-700D, we found that the *flrA* gene sustained a 49-bp internal deletion mutation (we hereafter refer this strain as GSΔ*flrA*) (**Figure 1**) causing a frame shift mutation. To determine if the 49-bp deletion in *flrA* gene in GSΔ*flrA* inhibited motility, we created an identical 49-bp deletion mutation in the wild-type *V. cholerae* strain N16961S. Data presented in **Figure 2** show that, in contrast to the wild-type *V. cholerae* N16961S strain (**Figure 2A**), the deletion mutation in the *flrA* gene in N16961S strain inhibited motility (**Figure 2B**). Furthermore, complementation of N16961SΔ*flrA* with the wild-type *flrA* gene cloned and expressed from pWSK29 (pAA146) restored motility to the level seen with the wild-type strain (**Figure 2C**). Our data clearly indicate that the potential frame shift mutation (49-bp deletion) in *flrA* gene had no effect on downstream genes in motility. In contrast, inhibition of motility seen with GSΔ*flrA* (**Figure 2D**) was only partially complemented by the same complementing vector (pAA146) (**Figure 2E**) indicating that GSΔ*flrA* might have additional mutation(s) affecting motility. As expected, the N16961SΔ*flaA* mutant was defective in motility (**Figure 2F**). In summary, our data confirmed that the deletion mutation of the *flrA* gene in GSΔ*flrA* and N16961S (N16961SΔ*flrA*) inhibited motility.

### FlrA and MshA of *V. cholerae* Inversely Promoted *vps*-Independent Biofilm in FSLW Microcosm

**FIGURE 3:**
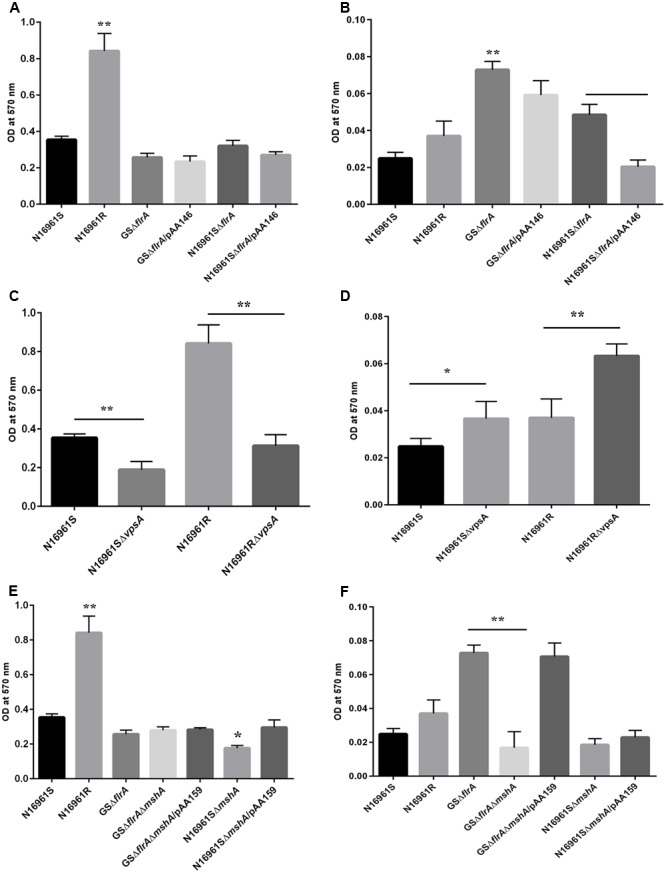
Quantitative measurement of biofilm produced by *V. cholerae* strain. Quantification of biofilm produced by *V. cholerae* strains grown in: nutrient-rich L-broth **(A,C,E)**, and in nutrient-poor FSLW **(B,D,F)**. The results represent the average of six independent experiments. All the values are expressed as mean ± Δstandard error (SE) calculated from the six readings for each strain. A *p*-value of <0.05 was considered statistically significant; ^∗∗^*p* < 0.01, ^∗^*p* < 0.05.

We previously reported that the GASP-700D phenotype produced *vps*-independent biofilm in FSLW microcosms with a ∼1,000-fold down regulation of *flrA* gene (Jubair et al., 2014). However, in that study, we were unable to elucidate which genetic elements, if any, were involved in such biofilm formation. Previous investigations have demonstrated that *V. cholerae* employs its flagella and mannose sensitive hemagglutinin (MSHA) pilus, in concert for *vps*-dependent biofilm formation (Watnick et al., 1999; Watnick and Kolter, 1999). To test if flagella and MSHA pilus contribute similarly to the formation of *vps*-independent biofilm, we compared biofilm-forming ability between a wild-type *V. cholerae* N16961S strain and its *flrA* and *mshA* mutants both in nutrient-rich L-broth and in nutrient-deficient FSLW. As a control, we used a *V. cholerae* rugose strain (N16961R) that produces copious amounts of exopolysaccharide and robust biofilm both in L-broth and in FSLW (Jubair et al., 2014). As depicted in **Figure 3A**, except for the *V. cholerae* rugose strain, none of the tested strains, including wild-type N16961S and *flrA* mutants (N16961SΔ*flrA* and GSΔ*flrA*) were able to produce significant biofilm in L-broth. In contrast, when grown in FSLW (**Figure 3B**), both GSΔ*flrA* and N16961SΔ*flrA* produced significantly (*p* < 0.01) higher amount of biofilm relative to the wild-type N16961S strain. Furthermore, complementation of N16961SΔ*flrA* with the wild-type *flrA* gene repressed biofilm formation (**Figure 3B**); however, complementation of GSΔ*flrA* with wild-type *flrA* gene was unable to significantly repress biofilm formation (**Figure 2B**) reinforcing our hypothesis that GSΔ*flrA* might have additional mutation(s) affecting motility and *vps*-independent biofilm formation. In summary, our results demonstrated that mutation in *flrA* gene in *V. cholerae* promoted increased biofilm formation specific to FSLW. Furthermore, we did not observe any significant changes in biofilm formation in FSLW among tested strains when biofilm was quantified after 48 h of incubation at room temperature (Supplementary Figure 1).

To determine if gene(s) required for *vps*-dependent biofilm formation enhances *vps*-independent biofilm formation both in L-broth and in FSLW, we compared biofilm-forming ability of *vpsA* mutants to that of corresponding wild-type strains both in L-broth and in FSLW. Data presented in **Figure 3C** exhibited that, as expected, in L-broth, *vpsA* mutants significantly inhibited *vps*-dependent biofilm formation compared to their respective wild-type variants; in contrast, *vpsA* mutants significantly increased biofilm production compared to their wild-type strains in FSLW (**Figure 3D**). In summary, consistent with our previous report (Jubair et al., 2014) we demonstrated that mutation in *vpsA* gene positively contributed to *vps*-independent biofilm in FSLW, but not in L-broth.

To determine the role for MSHA pilus, if any, for *vps*-independent biofilm production in FSLW, we created an in-frame deletion within the *mshA* gene both in GSΔ*flrA* and in the N16961S background resulting in GSΔ*flrA*Δ*mshA* and N16961SΔ*mshA*, respectively. Mutation in *mshA* gene in GSΔ*flrA* (GSΔ*flrAΔmshA*) had no effect on biofilm production in L-broth compared to its wild-type strain (**Figure 3E**), while N16961SΔ*mshA* significantly reduced (*p* < 0.05) biofilm formation relative to its wild-type strain N16961S (**Figure 3E**). In contrast to L-broth, mutation in the *mshA* gene in GSΔ*flrA* significantly (*p* < 0.01) repressed biofilm formation in FSLW (**Figure 3F**). Moreover, complementation of GSΔ*flrA*Δ*mshA* with the wild-type *mshA* gene cloned and expressed *in trans* from plasmid pAA159 completely restored biofilm formation as seen with GSΔ*flrA* in FSLW (**Figure 3F**). Wild-type *V. cholerae* strain N16961S and its isogenic *mshA* mutant (N16961SΔ*mshA*) exhibited no significant difference in biofilm formation in FSLW (**Figure 3F**). In summary, our results confirmed that, while FlrA negatively affects the expression of *vps*-independent biofilm production in FSLW, MSHA pilus positively contributes to *vps*-independent biofilm production elicited by GSΔ*flrA* in FSLW. Unlike Δ*flrA*, a strain carrying *mshA* mutation in N16961S was unable to promote *vps*-independent biofilm formation in FSLW (**Figure 3F**).

**FIGURE 4:**
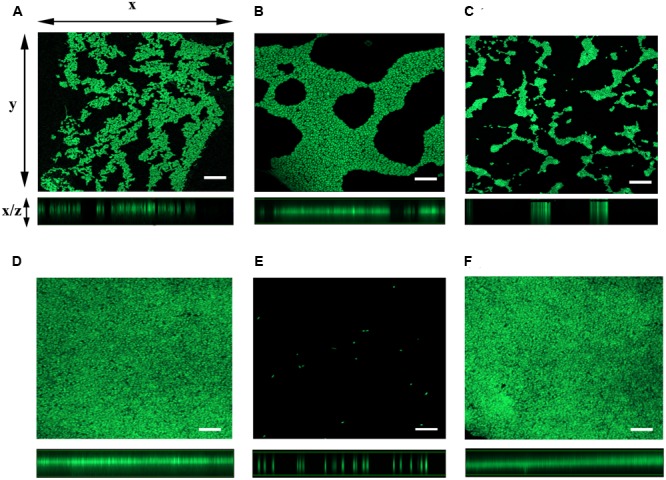
Analysis of three-dimensional architecture of *V. cholerae* biofilm using confocal microscope. Each strain of interest was grown in a 12-well culture plate containing 600 μl FSLW. A glass cover slip was dipped into each culture well and the cultures were incubated overnight statically at room temperature. SYTO 9 dye was added to the culture wells to stain the cover slip and the image was obtained using a laser scanning confocal microscope with an excitation and emission wavelengths of 484 and 500 nm, respectively. Images of *x*–*y* sections (top panels) of biofilm and *x*–*z* projections (bottom panels) of the same biofilm were captured and analyzed using ZEN 2 imaging software (Blue edition). **(A)** *V. cholerae* N16961S, **(B)** N16961SΔ*flrA*, **(C)** N16961SΔ*flrA*/pAA146, **(D)** GSΔ*flrA*, **(E)** GSΔ*flrA*Δ*mshA*, and **(F)** GSΔ*flrA*Δ*mshA*/pAA159; bar-10 μm.

To further examine the role for *mshA* gene in the expression of Δ*flrA*-driven biofilm in FSLW, we compared three-dimensional architectural structures and thickness of biofilm among wild-type N16961S, N16961SΔ*flrA*, N16961SΔ*flrA*/pAA146, GSΔ*flrA*, GSΔ*flrA*Δ*mshA*, and GSΔ*flrA*Δ*mshA*/pAA159 using confocal microscopic analysis. As expected, N16961Δ*flrA* produced more biofilm (**Figure 4B**) compared to wild-type N16961S (**Figure 4A**). Complementation of N16961Δ*flrA* with wild-type *flrA* gene (N16961SΔ*flrA*/pAA146) repressed biofilm formation (**Figure 4C**). In contrast to N16961Δ*flrA*, GSΔ*flrA* produced much more biofilm when grown in FSLW (**Figure 4D**). GSΔ*flrA*Δ*mshA* completely inhibited biofilm formation (**Figure 4E**); complementation of GSΔ*flrA*Δ*mshA* strain with the wild-type *mshA* gene fully restored biofilm production at the level seen with GSΔ*flrA* (**Figure 4F**). To rule out the possibility that the Δ*mshA* mutant may not have survived in FSLW, we enumerated microorganisms’ counts for each culture before making the slides used for confocal microscopic analysis using standard plate counts. Each culture yielded ca. 10^8^ cfu/ml suggesting that spotty occurrence of GSΔ*flrA*Δ*mshA* (**Figure 4E**) cells on slide resulted from the inability of the cells to form biofilm, and, not due to low cell counts present in the culture.

### *vps*-Independent Biofilm Promoted *V. cholerae* to Move toward Nutrients in FSLW

**FIGURE 5:**
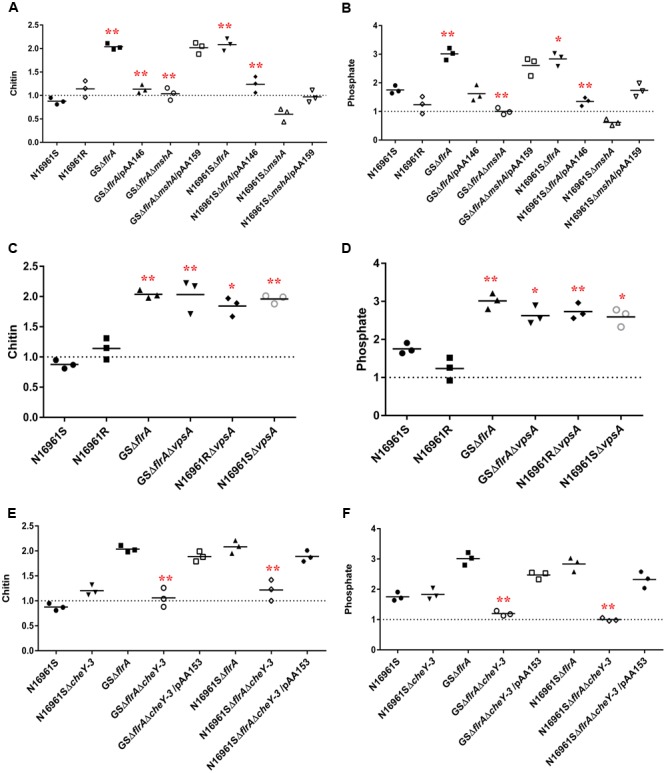
*vps*-independent biofilm promoted *V. cholerae* to move toward nutrient sources in FSLW as measured by chemotaxis. Chemotaxis assay was performed on *V. cholerae* strains as described previously (Butler et al., 2006) with modifications. Briefly, assay was performed in nutrient-deficient FSLW or FSLW supplemented with nutrients, including chitin or phosphate. The results represent three independent experiments and the data were expressed as a comparative index (CI) for each strain. CI was calculated by the number of bacterial counts (colony forming units per milliliter) present in FSLW in the capillary tube supplemented with nutrient divided by the number of bacterial counts (colony forming units per milliliter) present in FSLW in the capillary tube not supplemented with nutrient. The horizontal bar indicates the mean of the results from three capillary tube experiments. The observations resulting from an experiment pointing at or below the dashed line reflect no measurable response between control (-nutrient) and experiment (+nutrient) for each strain examined. A *p*-value of <0.05 was considered statistically significant. Impact of Δ*flrA* and Δ*mshA* mutants and reverted mutants on: **(A)** chitin and **(B)** phosphate; impact of Δ*cheY-3* mutants and reverted mutants on: **(C)** chitin and **(D)** phosphate; impact of Δ*vpsA* mutants on: **(E)** chitin and **(F)** phosphate; ^∗∗^*p* < 0.01,^∗^*p* < 0.05.

In aquatic environments, many planktonic bacterial species use a combination of chemotaxis and flagellar motility to sense and acquire nutrients (Butler and Camilli, 2005). *V. cholerae* is no exception to this phenomenon. However, it is not known how non-motile bacterial phenotypes or phenotypes lacking productive motility (commonly found in biofilm communities) acquire nutrients from their aquatic reservoir. Specifically, we questioned whether non-motile *V. cholerae*, promoting Δ*flrA*-driven *vps*-independent biofilm in FSLW, can sense and move toward nutrients and, if so, does chemotaxis play any role in such mechanism? In our previous study (Jubair et al., 2012), we reported that nutrients, including chitin and phosphate (when added to FSLW microcosm) promoted the growth of highly starved (180 and 700 days surviving microorganisms) *V. cholerae* persisting in nutrient-poor FSLW microcosms. Based on that observation we hypothesized that chitin and phosphate serve as chemo-attractants in chemotaxis assays in FSLW. As illustrated in **Figure 5**, GSΔ*flrA* and N1696S1*ΔflrA* mutants moved toward chitin (**Figure 5A**) and phosphate (**Figure 5B**) compared to their wild-type phenotype (N16961S). Strikingly, a *V. cholerae* rugose strain (N16961R), capable of producing robust *vps*-dependent biofilm (Ali et al., 2002), was unable to move toward nutrients in FSLW (**Figures 5A,B**). Complementation of GSΔ*flrA* and N1696S1*ΔflrA* with a wild-type *flrA* gene inhibited movement to nutrients (**Figures 5A,B**). In contrast and, as expected, GSΔ*flrA*Δ*mshA*, that repressed *vps*-independent biofilm (described above), was unable to move toward nutrients (**Figures 5A,B**). Not surprisingly, unlike the N16961SΔ*flrA* mutant, the N16961SΔ*mshA* mutant, that was unable to induce *vps*-independent biofilm, failed to move to nutrients (**Figures 5A,B**).

We previously reported that *vpsA* mutants inhibiting *vps*-dependent biofilm promoted *vps*-independent biofilm in FSLW (Jubair et al., 2014). We questioned if Δ*vpsA*-driven biofilm can also move toward nutrients in FSLW. To test our idea we compared the ability of *vpsA* mutants to move toward nutrients relative to their corresponding wild-type strains. Data presented in **Figures 5C,D** indicated that Δ*vpsA*-driven biofilm akin to Δ*flrA*-driven biofilm moved to chitin and phosphate compared to their corresponding wild-type strains. Our data clearly indicate that in contrast to *vps*-dependent biofilm, *vps*-independent biofilm elicited by both Δ*flrA*- and Δ*vpsA*-driven biofilm can promote *V. cholerae* to move toward nutrients, including chitin and phosphate in FSLW.

Previous reports suggested that bacterial chemotaxis genes, including *cheY-3* are involved in chemotaxis (Lee et al., 2001). To determine if *cheY*-3 plays any role to promote *V. cholerae* to move toward nutrients in FSLW, we created a null mutation in *cheY-3* gene in the background of N16961S, GSΔ*flrA*, and N16961SΔ*flrA*. As depicted in **Figures 5E,F**, compared to wild-type strain N16961S, GSΔ*flrA*Δ*cheY-3* and N16961SΔ*flrA*Δ*cheY-3* significantly (*p* < 0.01) repressed their movement toward chitin and phosphate; complementation of mutation with the wild-type *cheY*-3 gene restored movement to chitin and phosphate (**Figures 5E,F**). Interestingly, we observed that there was no significant difference in biofilm production between N16961SΔ*flrA* and N16961SΔ*flrA*Δ*cheY-3* (Supplementary Figure 2). Our data indicate that CheY-3 of *V. cholerae* significantly (*p* < 0.01) contributed to movement toward nutrients, including chitin and phosphate in FSLW.

## Discussion

*Vibrio cholerae* produces two types of biofilm, including *vps*-dependent and *vps*-independent biofilms (Kierek and Watnick, 2003a,b; Moorthy and Watnick, 2004). *vps*-dependent biofilm, encoded by *vps* gene clusters, promotes “rugose” colony morphology associated with three-dimensional biofilm matrix consisting of pillars interspersed with water channels (Yildiz and Schoolnik, 1999). The formation of *vps*-dependent biofilm has been investigated in poorly defined or undefined commercially available media, including L-broth containing monosaccharides, aminoacids, and vitamins among other ingredients (Ali et al., 2002; Beyhan and Yildiz, 2007). In addition to undefined media, *vps*-dependent biofilm has also been studied in minimal media supplemented with monosaccharides (Wai et al., 1998; Moorthy and Watnick, 2004). In contrast to undefined media or defined media contributing to *vps*-dependent biofilm formation, previous studies (Kierek and Watnick, 2003a,b) have demonstrated that only *vps*-dependent biofilm, not *vps*-independent biofilm, can be induced in fresh surface water supplemented with monosaccharides. In contrast, *V. cholerae* produced both *vps*-dependent and *vps*-independent biofilm in commercially available artificial seawater (ASW) or real seawater supplemented either with monosaccharide sources (yeast extract) or vitamin assay casamino acids (CAA), also referred as defined media (Kierek and Watnick, 2003b,a; Moorthy and Watnick, 2004). Furthermore, early investigations have reported that Ca^+2^ ions of seawater contributed to *vps*-independent biofilm, and that *V. cholerae* flagella and MSHA pilus are required in concert for *vps*-independent biofilm as seen with *vps*-dependent biofilm (Kierek and Watnick, 2003b).

Contrary to previous investigations described above, we observed that, compared to wild-type *V. cholerae* N16961S strain, mutation in the *flrA* gene in *V. cholerae* promoted *vps*-independent biofilm formation in FSLW, not supplemented with any extraneous nutrients (**Figures 3B, 4B**). In addition to the Δ*flrA*, inactivation of *vpsA* (**Figures 3C,D**; Jubair et al., 2014), inhibiting *vps*-dependent biofilm formation, promoted *vps*-independent biofilm in FSLW. In summary, our current observation and a previous report (Jubair et al., 2014) suggest that *vps*-independent biofilm is produced in FSLW either by inactivation of *flrA* or *vpsA*. Our observation is in contrast to a previous study (Kierek and Watnick, 2003a) indicating that a *vps* mutant was unable to form *vps*-independent biofilm in fresh water even with supplementation of nutrients. The observed differences between our study and that previous report (Kierek and Watnick, 2003a) could be attributed to *V. cholerae* strain differences: while we used *V. cholerae* N16961S strain (a serogroup O1 biotype El Tor strain), previous studies used a *V. cholerae* MO10 strain (a serogroup O139 strain possessing a capsule unlike serogroup O1 strain). Indeed, *V. cholerae* strains with distinct genetic and phenotypic traits exhibited distinct *vps*-dependent biofilm formation as reported previously (Watnick et al., 2001; Ali et al., 2002). We primarily used GSΔ*flrA* and N16961SΔ*flrA* for this study. In contrast to N16961SΔ*flrA*, we were only able to partially restore motility and biofilm phenotypes in GSΔ*flrA* using a complementing vector implying that GSΔ*flrA* may have sustained additional mutation(s) affecting these phenotypes. In future studies, we will perform whole-genome sequencing and bioinformatic analysis to determine if GSΔ*flrA* indeed sustained additional mutation(s). Consistent with a previous study (Kierek and Watnick, 2003b), we found that MSHA is required for Δ*flrA*-driven *vps*-independent biofilm formation in FSLW (**Figures 3F, 4E**).

In *vps*-dependent mature biofilm, *V. cholerae* inhibits flagella synthesis while promoting VPS biosynthesis; thus, it is tempting to speculate that *vps*-independent biofilm could be a component of the *vps*-dependent mature biofilm. Indeed, previous studies have demonstrated that both *vps*-dependent and *vps*-independent biofilm are produced in real seawater and ASW supplemented with nutrients and vitamins (Kierek and Watnick, 2003a,b). Given the very high energy cost associated with the production of exopolysaccharide and biofilm, why would *V. cholerae* prefer to synthesize two types of biofilm at a given time? Does *V. cholerae* alternate between *vps*-dependent and -independent biofilm pathways after sensing environmental cues requiring a specific biofilm formation pathway? These are important questions that are yet to be fully elucidated. However, available data suggest that *V. cholerae* employs *vps*-dependent biofilm to confer resistance to chlorine, oxidative and osmotic stresses (Morris et al., 1996; Wai et al., 1998; Yildiz and Schoolnik, 1999); furthermore, this biofilm protects the bacterium from predators (Matz et al., 2005). Based on these studies, it has been inferred that *vps*-dependent biofilm promotes *V. cholerae*’s environmental persistence by evading environmental stressors. So, what is the role of *vps*-independent biofilm? In our recent study (Jubair et al., 2014), we reported that *vps*-independent biofilm resists only oxidative stress, not chlorine or osmotic stresses. Furthermore, we demonstrated that *vps*-independent biofilm is scattered and dispersed in nature (Jubair et al., 2014). Based on these observations, we hypothesized that *vps*-independent biofilm attracts or scavenges scarce nutrients present in aquatic reservoirs. Our hypothesis is based in part of our earlier finding (Jubair et al., 2012) that very starved and aggregated *V. cholerae* (identified as a novel “persister” phenotype) acquired chitin and phosphate from FSLW microcosms supplemented with these nutrients. Consistent with our hypothesis we have demonstrated that strains having *vps*-independent, but not *vps*-dependent, biofilm moved toward nutrients, including chitin and phosphate, as determined by a standard chemotaxis assay (**Figures 5A,B**). Intriguingly, strains with *vps*-independent biofilm resulting from either inactivation of *flrA* or *vpsA* were able to move toward nutrients in FSLW suggesting that *V. cholerae* has retained flexibility in attracting nutrients (**Figures 5A–D**). For example, the microorganism can retain functional *vps*-dependent biofilm while promoting *vps*-independent biofilm by repressing *flrA*. Alternatively, the bacterium may repress *vps* genes facilitating down-regulation of *vps*-dependent biofilm while promoting *vps*-independent biofilm without affecting *flrA* (retain motility). Generally, bacterial motility and chemotaxis are interdependent or work in concert (Butler and Camilli, 2005); however, we report here that non-motile Δ*flrA*-driven *vps*-independent biofilm promotes movement of *V. cholerae* toward nutrients. Currently, we do not have evidence to show how non-motile bacterium moves to nutrients; however, we speculate that non-motile cells move as biofilm (aggregate) toward nutrient. As *vps*-independent biofilm is *mshA* dependent and *vpsA* independent, it is possible that biofilm matrix deposition (adhesin) on the microorganism’s cell surface and/or bacterial cell–cell interaction via MSHA pilus contribute to the movement of biofilm bacteria toward nutrients.

*Vibrio cholerae* is ubiquitous to aquatic environments where it can persist for decades. Microorganism can use chitin, the most abundant source of carbon in aquatic reservoirs (Dang and Lovell, 2016), as a sole carbon source using genetic elements present in the bacterium’s chromosome (Meibom et al., 2004). However, to maintain optimal growth, bacteria require balanced nutrients; unfortunately, inorganic phosphate, a key growth-promoting factor for *V. cholerae*, is very limited or absent in both fresh and ocean water limiting the optimal growth of bacteria, including culturable forms of toxigenic *V. cholerae* (Smith and Schindler, 2009; Schindler, 2012). Because of nutrient limitation, the isolation of culturable *V. cholerae* from aquatic reservoirs has been very low even during ongoing outbreaks in a country where cholera is epidemic (Huq et al., 2005; Alam et al., 2015). However, available data suggest that agricultural runoff, particularly during rainfall, enriches surface and estuary waters with increased inorganic phosphate (Smith and Schindler, 2009). Based on the data presented in this paper we propose that strains expressing the *vps-*independent biofilm of toxigenic *V. cholerae* are able to move to acquire phosphate promoting the bacterium’s growth at least transiently in aquatic reservoirs. Consumption of water with increased toxigenic *V. cholerae* counts following rainfall may serve as a trigger for cholera epidemics, in keeping with the observation that epidemics often follow rainfall events in cholera endemic countries (Alam et al., 2006, 2015).

In summary, we provide evidence in this paper that *vps*-independent biofilm plays a major role in attraction to nutrients in FSLW. Our observations could have major implications for our understanding of the persistence of *V. cholerae* in aquatic reservoirs, the potential impact of nutrient (phosphate and chitin) recycling (Dang and Lovell, 2016), and factors responsible for initiation of cholera epidemics in cholera-endemic countries.

## Author Contributions

AA designed and supervised the research experiments; SS-R performed the experiments and processed the data; AA and SS-R interpreted data and performed statistical analysis; and AA and SS-R wrote, critically revised, and approved the manuscript.

## Conflict of Interest Statement

The authors declare that the research was conducted in the absence of any commercial or financial relationships that could be construed as a potential conflict of interest.
