# Supplementary material for: Mutation in flrA and mshA Genes of Vibrio cholerae Inversely Involved in vps-Independent Biofilm Driving Bacterium Toward Nutrients in Lake Water
Source: Front Microbiol. 2017 Sep 13;8:1770. doi: 10.3389/fmicb.2017.01770 (PMC5604084; doi:10.3389/fmicb.2017.01770)
Supplement: Supplementary file 1 [file Table_1.DOCX]

Supplementary Material

Mutation in *flrA* and *mshA* Genes of *Vibrio cholerae* Inversely Involved in *vps*-independent Biofilm Driving Bacterium toward Nutrients in Lake Water

Shrestha Sinha Ray and Afsar Ali^*^

*** Correspondence:**

Afsar Ali
[aali@epi.ufl.edu](mailto:aali@epi.ufl.edu)

# Supplementary Tables and Figures

## Supplementary Tables

**Supplementary Table 1. Oligonucleotide primers used in this study.**

| Primer name | Direction | PCR step | Gene | Sequence (5’ to 3’)^a^ | Reference |
| --- | --- | --- | --- | --- | --- |
| aa778 | F | N/A  *flrA* | | CCT ATG CAG AGT TTA GCG | This study |
| aa780 | R | N/A  *flrA* | | CTA GCG TTG CAT GTT  GTA | This study |
| aa778S | F^c^ | N/A *flrA* | | AAA AGT CGA CCC TAT GCA GAG TTT AGC G | This study |
| aa780S | R^d^ | N/A *flrA* | | AAA AGA GCT CCT AGC GTT GCA TGT TGT A | This study |
| aa472 | F^c^ | First *cheY-3* | | CCG CGG CTT AGA ACT TGT CGC CTG C | This study |
| aa473 | R |  |  | GC TCT AGA CCT CCA CTG AGT TTG AGA TCA G | This study |
| aa474 | F | Second *cheY-3* | | GC TCT AGA GTG CTC TAT TCA CAC GCG C | This study |
| aa475 | R^d^ |  |  | CG GGA TCC GCA GGT CTC CAC TAG CTC G | This study |
| aa752 | F | First *mshA* | | AAA AGT CGA CGC GAA AGC GAA TAG TGG | (Thelin and Taylor, 1996) |
| aa753 | R |  |  | AAA AGG ATC CAT TGC ACC AGC AAC TGC ACC | (Thelin and Taylor, 1996) |
| aa754 | F | Second *mshA* | | AAA AGG ATC CTG CAA CGG TTG CTA TGC | (Thelin and Taylor, 1996) |
| aa756 | R |  |  | AAA AGA GCT CGT GGT TAC CAC CGC AAA GG | (Thelin and Taylor, 1996) |

^a^ Underlined sequences represent restriction sites

F, Forward Primer

R, Reverse Primer

^c,d^ Forward and reverse primers used for complementation of target gene respectively.

## Supplementary Figures





**Supplementary Figure 1. Comparison of biofilm production between wild-type and its respective mutant *V. cholerae* strains grown at 24 and 48 h in nutrient-poor FSLW**. The biofilm assay was carried out as described previously (Jubair et al., 2014). The results represent the average of six independent experiments. All the values are expressed as mean ± standard error (SE) calculated from the six readings for each strain.

**

Supplementary Figure 2.** **Comparison of biofilm production between wild-type *V. cholerae* and its corresponding Δ*cheY*-3 mutant strains in filter-sterilized lake water (FSLW).** Quantification of biofilm production by *V.cholerae* strains grown in nutrient-poor FSLW was carried out as described previously (Jubair et al., 2014). The results represent the average of six independent experiments. All the values are expressed as mean ± standard error (SE) calculated from the six readings for each strain; *P<0.05, ns is not significant.

**Reference**

Jubair, M., Atanasova, K.R., Rahman, M., Klose, K.E., Yasmin, M., Yilmaz, O., et al. (2014). *Vibrio cholerae* persisted in microcosm for 700 days inhibits motility but promotes biofilm formation in nutrient-poor lake water microcosms. *PloS ONE* 9(3).

Thelin, K.H., and Taylor, R.K. (1996). Toxin-coregulated pilus, but not mannose-sensitive hemagglutinin, is required for colonization by *Vibrio cholerae* O1 El Tor biotype and O139 strains. *Infection and Immunity* 64**,** 2853-2856.
